# Integrating Chemical Ecology with Behavioural Bioassays to Understand Host Preferences in the Stink Bug Egg Parasitoids *Trissolcus basalis* and *Trissolcus oenone* (Hymenoptera: Scelionidae)

**DOI:** 10.1007/s10886-025-01616-z

**Published:** 2025-05-28

**Authors:** Thomas E. Saunders, Lee-Anne M. Manning, Kye Chung Park, Gregory I. Holwell, Gonzalo A. Avila

**Affiliations:** 1 Te Kura Mātauranga Koiora, School of Biological Sciences, Waipapa Taumata Rau, University of Auckland, Private Bag 92019, Auckland, New Zealand; 2 The New Zealand Institute for Plant & Food Research Limited, Auckland, New Zealand; 3 Better Border Biosecurity (http://www.b3nz.org), Auckland, New Zealand

**Keywords:** Host Range, Chemo-orientation, Scelionidae, Pentatomidae, Biocontrol

## Abstract

Current approaches to assessing potential non-target risks associated with biological control agents are conservative, and they often rely on oviposition experiments conducted in quarantine laboratories. By their nature, such tests offer robust evidence of a parasitoid’s ability to attack and develop in a host. However, they exclude many important chemical cues present in the natural environment, which play a key role in the ability of a parasitoid to search for and locate hosts. We conducted a series of experiments with *Trissolcus basalis* and *Trissolcus oenone* to better understand the chemical basis mediating differences in host-specificity between these parasitoids. First, we compared the searching behaviour of *T. basalis* and *T. oenone* in open arena arrestment bioassays contaminated with footprint compounds of *Nezara viridula* or *Cuspicona simplex*. *Trissolcus basalis* spent four times longer searching for *N. viridula* than *C. simplex*, while *T. oenone* spent four times longer searching for *C. simplex* than *N. viridula*. We then conducted competition experiments to assess factors important to determining the outcomes of extrinsic and intrinsic contests between these parasitoids when they are simultaneously exposed to *C. simplex* egg masses. *Trissolcus oenone* was the superior competitor in extrinsic and intrinsic contests. Finally, we recorded the antennal responses of *T. basalis* to egg extracts of *N. viridula*, to tentatively identify potential contact kairomones used by this parasitoid to recognise and accept hosts. We discuss our results in the context of combining behavioural and chemical ecological techniques for pre-release risk assessments of classical biological control agents.

## Introduction

Parasitoids are widely used as classical biological control agents because they are often highly effective at finding and killing their hosts (Vinson 1998). Before a parasitoid can be released, it usually needs to be tested for the potential risks it may pose to non-target species (Barratt 2011). Scientific frameworks for host range testing emphasise a risk-based approach, beginning with measuring physiological host range through the use of no-choice tests and choice tests, before proceeding to field cage tests (Van Driesche et al. 2004; Bigler et al. 2006; van Lenteren et al. 2006). However, newer approaches to biological control such as pre-emptive/proactive programmes emphasise the need to find a suitable agent before the target pest has established (Hoddle et al. 2018). In these cases, researchers are unlikely to have approval to import the target pest and include it in detailed behavioural experiments. Regulators must therefore rely on the results from physiological host range testing in containment, which is necessarily more constrained in its testing regimes (Van Driesche and Murray 2004). No choice oviposition tests are an important first step in host-specificity testing as they provide unambiguous evidence of a parasitoid’s physiological (= fundamental) host range (Murray et al. 2010). But by their nature, physiological host range experiments are only designed to evaluate the final stages of the host-location process (Van Driesche et al. 2004). There is increasing interest in how the chemical basis of attraction between biological control agents and their hosts may affect both the performance of agents, and their ability to detect and attack non-target species (Conti et al. 2004; Salerno et al. 2006; Park et al. 2018).

Parasitoids rely on olfaction to detect compounds associated with their hosts and the broader environment, enabling them to locate and discriminate between hosts and non-hosts (Vinson 1998; Wajnberg and Colazza 2013; Colazza et al. 2014). Host semiochemicals therefore play a crucial role in the expression of host preferences. Classical biological control agents could be more effectively screened for non-target risks before release if we had a better understanding of how semiochemicals influence host preferences in the relevant study system (Salerno et al. 2006; Park et al. 2018). Integrating the results of chemical-ecological experiments with oviposition tests is a promising approach to providing more thorough analyses of host ranges and preferences during pre-release risk assessments conducted in containment. For example, arrestment experiments in open arenas demonstrate parasitoid attraction to blends of volatiles left behind on a substrate by a potential host, and can also show that parasitoids are able to discriminate between different hosts, and even different sexes or developmental stages (Colazza et al. 1999, 2007; Peri et al. 2006; Salerno et al. 2019). However, arrestment studies can also be used to compare the strength of attraction between different hosts (Conti et al. 2004). This information could be used to rank non-target hosts, based on how motivated a candidate agent is to search for them, and these results could be compared to the target host, if available. The length of time the parasitoid spends searching for a host could be used as a proxy for the motivation a parasitoid would have to seek out this host in the field. Another example is the integration of electrophysiological techniques to identify kairomones which may play a role in the host-specificity of biocontrol agents (Park et al. 2001; Iacovone et al. 2016; Li et al. 2017). Comparing bioactive volatile profiles between different hosts which elicit varying levels of searching motivation during arrestment experiments offers important clues as to which compounds or blends are acting as kairomones.

Intraguild competition between candidate biological control agents and resident parasitoids is rarely assessed during pre-release non-target risk assessments. The focus is usually on interactions between the candidate agent and a selection of non-target species. However, a greater appreciation of the ecological context in which a new agent is to be introduced should be investigated, if possible, to understand the interactions among parasitoids, and among multiple parasitoids and potential hosts. Interspecific competition between biological control agents and/or native parasitoids can occur both extrinsically (between females exploiting a host resource) and intrinsically (between larvae competing for the same host resources) ( Harvey et al. 2013; Cusumano et al. 2016). Both kinds of competition have important implications for the performance of biological control agents and the ecological dynamics of potential non-target attacks and their corresponding impacts. For example, female egg parasitoids typically run through a sequence of behavioural steps leading ultimately to oviposition or rejection of the egg (Colazza et al. 1999; Field and Keller 1999; Iacovone et al. 2016). During this process, parasitoids must decide between exploiting the host patch further, or instead defending it from intraguild intruders, who are capable of exploiting it even after the resident female has laid eggs and left the patch (Field and Calbert 1998; Vinson 1998). The way a biological control agent balances these interests has implications for their ability to exploit a host patch in the face of competition, and therefore the performance of the agent, the fitness of native parasitoids, and the distributions and survival of non-target species which may be attacked by both (Cusumano et al. 2012; Peri et al. 2014; Konopka et al. 2017).

Stink bugs (Hemiptera: Pentatomidae) are a diverse and widespread group, and some species are important pests in horticultural regions around the world ( McPherson 2018; Conti et al. 2021). Brown marmorated stink bug (*Halyomorpha halys* Stål), a serious horticultural pest native to East Asia, has recently spread through North America and Europe, causing millions of dollars of damage in lost crop yields (Leskey and Nielsen 2018; McPherson 2018). Natural enemy surveys in East Asia showed the egg parasitoid *Trissolcus japonicus* Ashmead (Hymenoptera: Scelionidae) is the most dominant natural enemy against BMSB, and it has therefore been the subject of host range testing in the United States since 2007 (Rice et al. 2014; Buffington et al. 2018), in New Zealand since 2015 (Charles et al. 2019; Saunders et al. 2021), and more recently in Europe (Haye et al. 2020). Egg parasitoids in the family Scelionidae are commonly employed as biological control agents against pentatomid pests (Orr 1988; Austin et al. 2005). Green vegetable bug, *Nezara viridula* (L.), is one of the most widespread crop pests in the world (Todd 1989). *Trissolcus basalis* (Wollaston) is the dominant natural enemy of *N. viridula*, being closely associated with it in Europe, the Americas, the Middle East, South Asia, and deliberately introduced for the purpose in Hawaii, Australia, New Zealand, and other parts of the Pacific (Jones 1988; Clarke 1990; Colazza and Bin 1995). In addition, research on the chemical ecology of the interaction between *N. viridula* and *T. basalis* has revealed important insights into the host selection and host location processes used by egg parasitoids (Bin et al. 1993; Colazza et al. 1999).

The chemical ecology of stink bug parasitoid host-ranges, and potential competition arising between introduced and native parasitoids, are currently of interest in New Zealand where *Trissolcus japonicus* Ashmead has been conditionally approved for release against BMSB, should the bug establish in New Zealand (EPA 2018). The Environmental Protection Authority made its decision on the basis of physiological host range testing against non-target Pentatomidae (Charles et al. 2019), the results of which were subsequently updated to include the endemic alpine shield bug, *Hypsithocus hudsonae* Bergroth (Saunders et al. 2021). Complementary work has since provided a more holistic overview of the physiological host ranges of the two resident pentatomid egg parasitoids: *Trissolcus basalis* (introduced against *N. viridula* in 1949); and *Trissolcus oenone* (Dodd) (native to New Zealand and Australia) (Saunders et al. 2022). In short, all three parasitoids attack and develop in all Pentatomidae at parasitism efficiencies exceeding 60%, with the exception of the lack of association of *T. japonicus* and *T. oenone* with *N. viridula*, and an untested association between *T. oenone* and *H. hudsonae*. Electrophysiology work revealed that New Zealand pentatomid volatile profiles largely overlapped qualitatively, but differed quantitatively, and that 7 shared compounds elicited antennal responses from *T. basalis*, *T. oenone*, and *T. japonicus* (Saunders et al. 2024). The potential future introduction of *T. japonicus* into New Zealand, along with the overlapping host-ranges of these three parasitoids, have stimulated interest in characterising potential ecological interactions between them (Todd et al. 2020), and investigating the chemical ecology linking them to their hosts.

The objective of our work was to use this parasitoid-host complex to evaluate the utility of integrating behavioural and chemical-ecological methodologies for insights into host preferences. We assess whether the combined results from arrestment, competition, electrophysiology, and oviposition tests provide insights relevant to host range testing which are greater than the sum of their parts. We focussed on the two resident parasitoids, *T. oenone* and *T. basalis*, and how they react to *N. viridula* (the primary host of *T. basalis*) and *Cuspicona simplex* Walker (Hemiptera: Pentatomidae) (a known host of both parasitoids). First, we conducted open arena arrestment bioassays with each parasitoid contaminated with footprint volatiles from each pentatomid species, and compared parasitoid retention time against uncontaminated control arenas. The purpose of these bioassays was to identify whether or not these parasitoids could discriminate between different hosts based solely on footprint kairomones, and to determine the strength of attraction between each parasitoid and pentatomid. Next, we conducted competition experiments by simultaneously releasing an individual female of each parasitoid species onto a *C. simplex* egg mass and recording behavioural interactions relevant to extrinsic competition. We also collected data on the outcome of larval contests in multiparasitised eggs to examine intrinsic competition between an introduced biological control agent and a native parasitoid. Finally, we conducted electrophysiological experiments whereby *T. basalis* was exposed to volatile extracts made with *N. viridula* eggs in order to compare responses between extracts made with hexane and those made with acetone. We also aimed to identify candidate chemical compounds which may act as contact kairomones facilitating host acceptance. Ultimately, olfactory cues play a major role in filtering physiological host range down to ecological host range, so a better understanding of how host chemistry mediates parasitoid behaviour can be used to improve pre-release host range testing and provide more certainty for decision makers.

## Methods

### Insect Rearing and Identification

Stink bug colonies were initiated with wild-collected specimens from around the Auckland region, and were housed in clear plastic containers measuring 170 mm × 210 mm × 135 mm with ventilated lids at 20–25 °C (16:8 h L: D) provisioned with moist cotton and fanned wax paper for oviposition substrate. *Solanum* fruits and tomatoes were provided as food for *C. simplex*, and green beans and raw peanuts for *N. viridula*. *Trissolcus basalis* was reared from parasitised *N. viridula* egg masses naturally laid on *Cleome* plants in Auckland, in February 2019. *Trissolcus oenone* was reared from parasitised *C. simplex* egg masses naturally laid on *Coprosma* (near *Solanum*) plants in Auckland, in November 2019. Parasitoids were reared at 20–25 °C (16:8 h L: D) on the eggs of the species they were originally collected in. Fresh eggs (< 24 h old), or eggs stored at 10 °C for no longer than 2 weeks, were used to maintain the colonies.

### Open Arena Arrestment Bioassays

We conducted arrestment bioassays with *T. basalis* and *T. oenone* to measure how long each parasitoid was arrested by the odours of adult *N. viridula* and *C. simplex*. Experiments were conducted in a 25°C temperature-controlled room lit with fluorescent lights, between the hours of 8am and 3pm. We used an open arena design inside a mesh cage measuring 59 cm L × 59 cm W × 59 cm D to prevent parasitoid escape. Arenas consisted of a plastic tray (46 cm L × 34 cm W) turned upside down with a piece of filter paper (21 cm diameter) on top. Each piece of filter paper had a 60 mm diameter circle in the centre of the paper. Five female stink bugs were taken from rearing cages and confined under a small petri dish inside the 60 mm circle for three hours prior to experiments. Single parasitoids were held in glass vials in the room for at least 30 minutes prior to experiments to acclimate. Stink bugs were subsequently removed from the paper and placed back into the colony. A single parasitoid was released into the centre of the filter paper by gently tapping the bottom of the vial while holding it upside down approximately 30 mm from the surface of the paper. Parasitoids which immediately flew away without walking on the paper at all were recaptured and discarded, including for control replicates, while parasitoids which remained on the paper for at least enough time to walk had their retention time recorded. We recorded the time parasitoids were retained in contact with the inner circle of the paper, which we defined as having ended once they moved approximately 5 mm past the inner line, as parasitoids were typically ‘pulled’ back into the circle during their searching as they zig-zagged around the circle. We also recorded the total time parasitoids remained in contact with the filter paper, which we defined as having ended once the parasitoid reached the outer margin of the paper or flew away. We also recorded whether or not the parasitoid displayed arrestment behaviour characteristic of scelionid egg parasitoids (described in Colazza et al. 1999). We recorded at least 30 replicates of each parasitoid exposed to each treatment.

### Competition Experiments

**Fig. 1:**
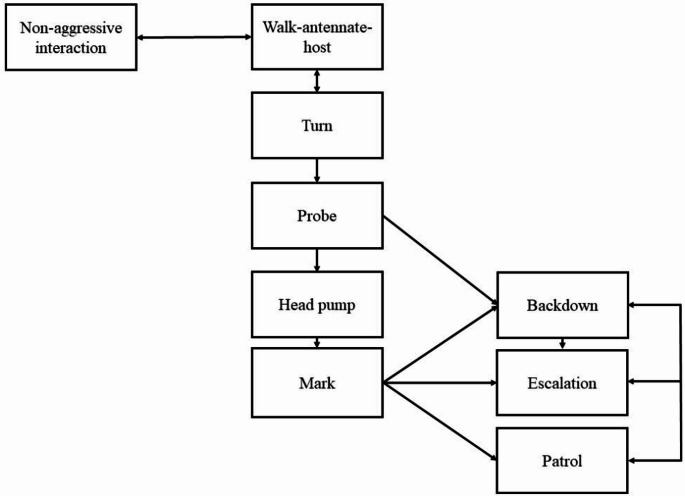
Ethogram showing generalised model of behaviours exhibited by parasitoids in competition experiments

We exposed *T. basalis* and *T. oenone* to *C. simplex* egg masses to observe the outcomes of aggressive encounters between single females from each species (extrinsic competition) and to record which species emerged from multiparasitised eggs (intrinsic competition). We mounted a fresh (< 24 h) *C. simplex* egg mass onto a 40 mm x 20 mm card with double sided sticky tape, and used white Scenic Sand (Activa Products) to cover any exposed tape. We placed the card into a plastic screw top vial (length 60 mm, diameter 28 mm), introduced a female parasitoid (the focal species, from which behavioural observations were recorded), and then screwed the lid back on. As soon as the focal species began oviposition, we immediately removed the lid and introduced a female parasitoid from the other species (the intruder), before replacing the lid. We placed the vial under a microscope fitted with an LED ring light to observe interactions between the two females. We recorded the time it took for the focal individual to make contact with the egg mass prior to introducing the intruder, how long it took for the focal individual to oviposit successfully in five eggs (indicated by marking the egg with her ovipositor), and the sequence of oviposition for both focal and intruder parasitoids. All oviposition events were recorded and each egg was individually identified within the mass with the aid of a diagram. We recorded either the total time taken for all eggs to have been oviposited in by both females, or the time taken until the parasitoids reached a ‘stalemate’ in their competition where one parasitoid guarded the egg mass and did not let the other approach. We also recorded the frequency and sequence of a variety of reproductive and agonistic behaviours exhibited by the focal species (Table 1; Fig. 1; behaviours modified from Field 1998). At the end of behavioural assays, the two parasitoids were removed and the pentatomid egg mass was kept for 10–11 days at 25 °C before freezing, just prior to parasitoid emergence. We did this in order to confirm the outcome of intrinsic contests by dissecting the eggs and identifying developing parasitoids.

**Table 1:** Behaviours observed during competition experiments with *Trissolcus basalis* and *Trissolcus oenone*. Modified from Field (1998).

Behaviour	Description
Walk-antennate-host	Walking over the egg mass while drumming antennae over the surface of the eggs.
Turn	Turns in a circle while standing on the top of a single egg, drumming the sides with antennae.
Probe	Extending wings, everting ovipositor, and then inserting ovipositor into an egg.
Pump	Pumping the head up and down during oviposition.
Mark	Sweeping the everted ovipositor in a figure of eight pattern over the surface of an egg straight after withdrawing ovipositor.
Non-aggressive interaction	An encounter between the parasitoids which didn’t result in agonistic behaviour, for example, brushing past each other, or inspecting one another at close range.
Patrol	Rapid movement over the surface of the egg mass and around the outside of the mass, often in response to perceiving a competitor in the vicinity.
Backdown	An aggressive encounter in which the aggressor makes physical contact with the receiver, at which point one of the parties immediately moves away from the other. Examples of contact include charging into the opponent, biting the wings, legs, antennae, or head of the opponent, or attempting to pull the opponent away from the egg it is currently ovipositing into.
Escalation	An extended aggressive encounter in which neither party backs down until after an intense physical contest, usually involving between five and twenty seconds of fighting.

### Extract Preparation and Chemical Analysis

We prepared stink bug egg solvent extracts with both acetone (Sigma-Aldrich, GmbH, HPLC grade, 99.9% purity) and n-hexane (Merck, Germany, organic trace analysis, 99.9% purity) to measure *T. basalis* antennal responses to compounds associated with eggs from its primary host, *N. viridula*. Egg extracts prepared with acetone are known to retain bioactive compounds responsible for eliciting oviposition behaviour in *T. basalis* (Bin et al. 1993). We also made a hexane extract to compare the chemical composition and strength of antennal responses with the acetone extract. For the acetone extract, we weighed out 0.8 g of eggs and immersed them in 2 ml of acetone in a glass vial for 96 h, before decanting the extract to a clean vial and freezing at -20 °C until required for analyses. For the hexane extract, we weighed out 0.25 g of egg masses and immersed them in 1 ml of hexane in a glass vial for 24 h, before decanting the extract to a clean vial and freezing at -20 °C until required for analysis. All egg masses used for each treatment were less than 48 h old, and this timing was chosen to balance the needs of maintaining insect colonies while also sourcing eggs for experiments. Each of the extracts were made with different immersion times because the original intention was to make three different immersion replicates for each solvent (24 h, 48 h, 96 h). This was ultimately not possible due to rearing and timing constraints. Each extract was analysed on a gas-chromatograph (GC, Agilent 7890B) coupled to a mass-spectrometer (MS, Agilent 5977 A). A 1 µl sample was injected into the GC in splitless mode and carried with helium gas at a flow rate of 1.6 mL/min. The GC column was non-polar (Agilent DB-5 ms) and measured 30 m × 0.25 mm ID × 0.25 μm film thickness. The temperature program started at 40 °C and was held for 2 min, then was increased to 250 °C at a rate of 4 °C/min, followed by a 10 degree per min ramp to 280 degrees, then held for 10 min. The transfer line was kept at 250 °C. Tentative identification of the compounds found in the extracts was done by comparison of the spectra with the mass spectral library (NIST MS Search version 2.4, 2020), and by Kovats’ indices.

### Electrophysiological Recordings

We recorded electroantennogram (EAG) responses to measure *T. basalis* antennal responses to *N. viridula* egg extracts made with acetone and hexane. We anaesthetised each female wasp with carbon dioxide gas before removing its head and the distal tip of one of its antennae with a fine scalpel under a stereomicroscope (Leica MZ16,). We positioned each specimen between two silver wire electrodes sheathed by glass capillaries pulled to fine points. Glass capillaries were trimmed with a ceramic cutter and filled with Ringer’s solution. The severed head was positioned into contact with the reference electrode and the severed antennal tip was positioned into contact with the recording electrode using a motorised micromanipulator (Sutter Instruments, USA). We concentrated each extract by 10× under a gentle stream of argon gas before applying a 10-µL aliquot to a 5 × 25 mm strip of filter paper (Whatman No. 1; Whatman, U.K.) and allowed the solvent to evaporate for 10s before placing the paper inside a glass Pasteur pipette (146 mm; Fisher Scientific Co., Pittsburgh, Pennsylvania) to form an odour cartridge. We inserted the tip of each odour cartridge into a 2 mm diameter hole in the glass airflow tube containing a charcoal-filtered and humidified air stream with a flow rate of 400 ml/min. The antennal preparation was positioned in front of the air stream. The recording electrode was connected to an amplifier (IDAC 4, Syntech, Germany) and we used Autospike software (v3.9, Syntech Research and Equipment, Hilversum, Netherlands) to record EAG responses. We wrapped the wide end of odour cartridge pipettes in aluminium foil when not in use to prevent evaporation of test compound, and we used each cartridge less than 10 times. For the control air cartridge, we kept the filter paper blank, and for the solvent control cartridges, we applied a 10-µL aliquot of neat hexane or acetone. For the standard stimulus cartridges, we applied 10 µg of (E)-2-decenal to the filter paper (as a 10-µL aliquot of 10 µg/10µl solution in hexane). We used a different parasitoid antenna for each recording and captured six recordings showing consistent responses for each extract with *T. basalis*. We started each recording with a puff of air, followed by 3 puffs each of hexane and acetone, as controls. The experimental portion of each recording consisted of a single puff of (*E*)-2-decenal, 3 puffs of the hexane or acetone extract, a single puff of (*E*)-2-decenal, 3 puffs of the other extract, and a single puff of (*E*)-2-decenal to finish. All puffs were spaced at intervals of 30 s.

### Data Analysis

Analyses were performed in R 4.0.2 (R Core Team 2020). For arrestment experiments, we tested if there were differences in retention times between the two parasitoids when exposed to volatiles of each pentatomid with a Poisson GLM, and we included the dates of experiments as a random effect. We calculated estimated marginal means and confidence intervals for each combination of arrestment treatment and back-transformed these onto the original scale to examine differences in retention time. For competition assays, we tested whether *T. basalis* and *T. oenone* differed in the number of oviposition attempts or time it took to successfully parasitise five eggs with two sample t-tests. We tested whether parasitoids differed in the number of times they initiated a ‘backdown encounter’ based on the number of eggs in the mass with a Poisson GLM, with ‘date’ as a random effect. We also tested whether there was a difference between the number of eggs each parasitoid species attacked before aggression was initiated, based on the number of eggs in each host patch, the number of offspring each parasitoid invested into the mass, or the time it took the focal species to successfully mark its first five eggs. For electrophysiological experiments, we tentatively identified compounds in *N. viridula* egg extracts by comparison of the compounds with the mass spectral library NIST Mass Spectral Search Program version 2.4, 2020. We also compared the Kovats retention index (KI) (Kováts and Weisz 1965) of each compound with a hydrocarbon series (C8 to C28) using the same temperature program and column type as the extracts. We normalised responses in EAG recordings in relation to EAG responses to a standard compound ((*E*)-2-decenal) made throughout each recording, and present mean responses to the two extracts from multiple recordings. The normalised EAG responses were subjected to ANOVA followed by Tukey HSD test for multiple comparison.

## Results

### Arrestment Bioassays

**Fig. 2:**
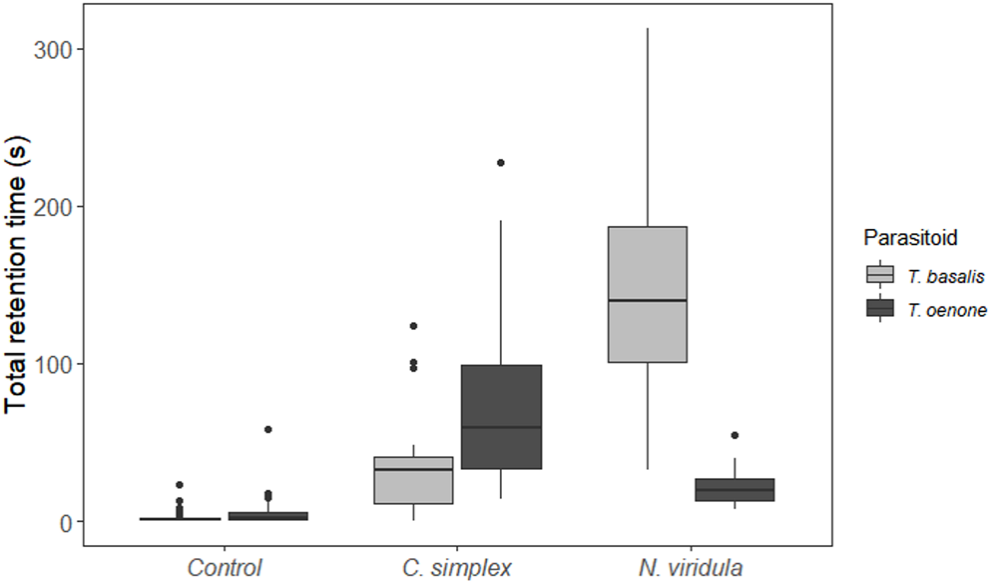
Boxplots showing mean retention times for *Trissolcus basalis* and *Trissolcus oenone* in open arenas contaminated with footprint compounds from *Cuspicona simplex* or *Nezara viridula*, compared to uncontaminated control arenas

*Trissolcus oenone* was arrested in a greater proportion of replicates for both pentatomid treatments than *T. basalis* (Fig. 2). Neither parasitoid showed any arrestment in control arenas. Parasitoids were significantly more likely (*F* = 66.286, df = 1, *P* < 0.001) to leave control arenas by flying, and contaminated arenas by walking to the edge of the paper.

Mean total retention time (the sum of inner and outer zones) differed based on a significant interaction between species of pentatomid and species of parasitoid (*F* = 63.76, df = 1, *P* < 0.001). *Trissolcus basalis* spent four times longer in arenas contaminated by *N. viridula* (mean = 149s, SD = 73s) than *C. simplex* (mean = 36s, SD = 33s), whereas *T. oenone* spent four times longer in arenas contaminated by *C. simplex* (mean = 80s, SD = 61s) than *N. viridula* (mean = 21s, SD = 11s) (Fig. 2). Mean retention times in the inner zone of the filter paper also differed based on a significant interaction between species of pentatomid and species of parasitoid (*F* = 63.5156, df = 1, *P* < 0.001). On average, both parasitoids spent the majority (75–82%) of their time in the inner zones of the filter paper contaminated by pentatomids. On average, both parasitoids spent less than 6 s in control arenas.

### Competition Experiments

In baseline oviposition experiments where there was no competition, *T. basalis* adults developed in (but did not emerge) or emerged from a mean of 97% (SD = 6%) of *C. simplex* eggs and 60% (SD = 34%) of *N. viridula* eggs, and this difference was significant (*F* = 7.4915, df = 1, < 0.01). *Trissolcus oenone* developed in or emerged from a mean of 90% (SD = 26%) of *C. simplex* eggs and 0% of *N. viridula* eggs. There was no significant difference in the percent emergence of the two parasitoids from *C. simplex* eggs (*F* = 0.8031, df = 1, *P* > 0.1).

In competition assays, *T. oenone* successfully oviposited in 18% more eggs than *T. basalis*, and developed in 94% of them (compared with T. basalis developing in only 25%). Just over half (51%) of eggs were oviposited into by both parasitoids, and of these, *T. oenone* developed in almost all of them (91%).

Behavioural sequences during competition experiments followed patterns shown in Fig. 1, with behaviours defined in Table 1. Frequencies of behavioural variables recorded during competition assays were similar for both parasitoid species. There was no difference between the two parasitoids in either the number of oviposition attempts (*t* = 1.6842, df = 18, *P* = 0.1094) or time it took to mark five eggs (*t* = 0.082254, df = 18, *P* = 0.9354), although *T. basalis* marked a higher proportion of eggs for each oviposition attempt (Table 2). *T. basalis* escalated fights more often than *T. oenone* (*F* = 51.632, df = 1, *P* < 0.001), and *T. oenone* was the receiver of more backdown agonistic encounters than *T. basalis* (*F* = 16.511, df = 1, *P* < 0.001). The number of eggs attacked by parasitoids before the first aggressive encounter did not significantly differ based on the parasitoid species, the number of oviposition attempts required for the parasitoid to mark five eggs, or the number of eggs in the mass.

**Table 2:** Mean frequencies of different behaviours observed during competition experiments between *Trissolcus basalis* and *Trissolcus oenone* on *Cuspicona simplex* eggs

Parasitoid	Marks per probe	SD	Walk-Antennate	SD	Probe	SD	Pump	SD	Mark	SD	Patrol	SD	Non-Agonistic	SD	Backdown (Aggressor)	SD	Backfown (Reciever)	SD	Escalation (Aggressor)	SD	Escalate (Reciever)	SD
*T. basalis*	0.74	0.10	28.50	11.59	20.60	6.70	16.40	4.79	15.10	4.91	11.70	3.74	6.80	8.75	2.20	2.20	6.80	5.71	15.60	17.55	3.40	2.88
*T. oenone*	0.66	0.18	31.20	17.76	25.90	15.27	19.00	10.77	15.10	3.67	12.20	1.69	3.60	4.45	1.20	2.04	12.50	15.69	4.80	9.22	2.00	2.58

### Chemical Analysis and Electrophysiology

We detected nine compounds in the hexane extract, 35 in the acetone extract, and of these only two (tetracosane and tricosane) were common to both (Table 3). Based on relative percentage, the most dominant compounds in the acetone extract were oleic acid, n-hexadecanoic acid, and octadecanoic acid. The compounds extracted from eggs using hexane were predominantly alkane hydrocarbons.

**Table 3:** Compounds tentatively identified in acetone and hexane extracts of *N. viridula* eggs based on NIST library matches

Solvent	Compound	KI	Relative %
acetone	5-methyl-3-Hexen-2-one	897	0.1
acetone	methoxy-phenyl-oxime	899	0.3
acetone	3-(methylthio)propionaldehyde	905	0.3
acetone	2-Hydroxy-2-cyclopenten-1-one	930	0.0
acetone	4-nitrophthalamide	940	0.0
acetone	DL-pantolactone	1040	0.1
acetone	benzeneacetaldehyde	1045	0.3
acetone	1-(2-methyl-1-cyclopentenyl)-ethanone	1099	0.1
acetone	catechol	1188	1.4
acetone	1,3-dimethylpentalongin	1256	0.2
acetone	ethyl (2’-nitro-3’-oxo-6’,7’-dihydro-3*H*,5*H*-benzo[ij]quinolizin-1’-yl)-cyanoacetate	1294	0.0
acetone	(+/-)-gamma-muurolene	1431	0.3
acetone	1-[2-[[(1,1-*D*imethylethyl)imino]methyl]-3-furanyl-1-propanone	1546	0.0
acetone	trans-Z-alpha-bisabolene epoxide	1599	0.2
acetone	dodecyl acrylate	1692	0.0
acetone	methyl hexadecanoate	1918	0.1
acetone	(Z)-11-hexadecenoic acid	1934	1.0
acetone	n-hexadecanoic acid	1959	13.6
acetone	1-methoxy-9-octadecene	2081	0.5
acetone	methyl oleate	2095	0.4
acetone	oleic acid	2147	64.3
acetone	octadecanoic acid	2163	6.6
acetone	(Z)-9-octadecenoic acide	2199	0.1
acetone	(*Z*)-9-octadecen-4-olide	2293	0.4
acetone	tricosane	2300	0.4
acetone	tetracosane	2400	0.7
acetone	pentacosane	2500	1.1
acetone	hexacosane	2600	0.9
acetone	(E)-9-octadecenoic acid pentyl ester	2698	2.5
acetone	5-butyl docosane	> 2700	1.7
acetone	bis(2-ethylhexyl) decanedioate	> 2700	0.6
acetone	3-methylheptacosane	> 2700	0.5
acetone	(1*R*, 2*S*)-2-(*H*ydroxymethyl)spiro[cyclopropane-1,3’-indol]-2‘(1’*H*)-one	> 2700	0.4
acetone	E-6-octadecen-1-ol acetate	> 2700	0.4
acetone	didecyl decanedioate	> 2700	0.1
hexane	9-(methylthio)-8 H-acenaphtho[1,2-c]pyrrole-7-carboxylic acid	976	0.9
hexane	tricosane	2300	2.7
hexane	tetracosane	2400	7.8
hexane	pentacosane	2500	15.4
hexane	hexacosane	2600	20.4
hexane	heptacosane	2700	20.3
hexane	octacosane	> 2700	12.8
hexane	nonacosane	> 2700	14.5
hexane	triacontane	> 2700	5.2

**Fig. 3:**
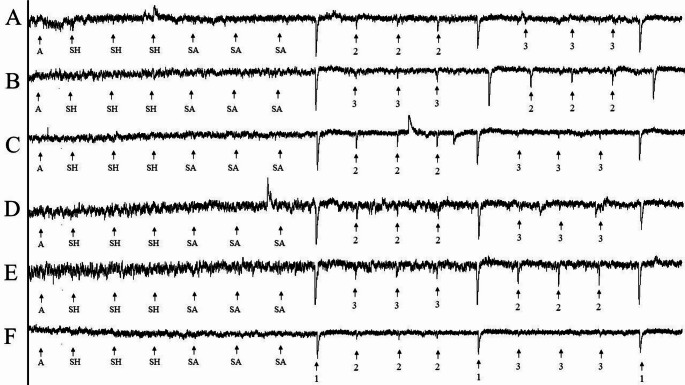
Electroantennogram experiments (**A**-**F**) showing responses of *Trissolcus basalis* to air (A), hexane (SH), acetone (SA), (E)-2-decenal (standard stimulus; 1), and sets of three puffs of *Nezara viridula* egg extracts made with acetone (2) and hexane (3)

**Fig. 4:**
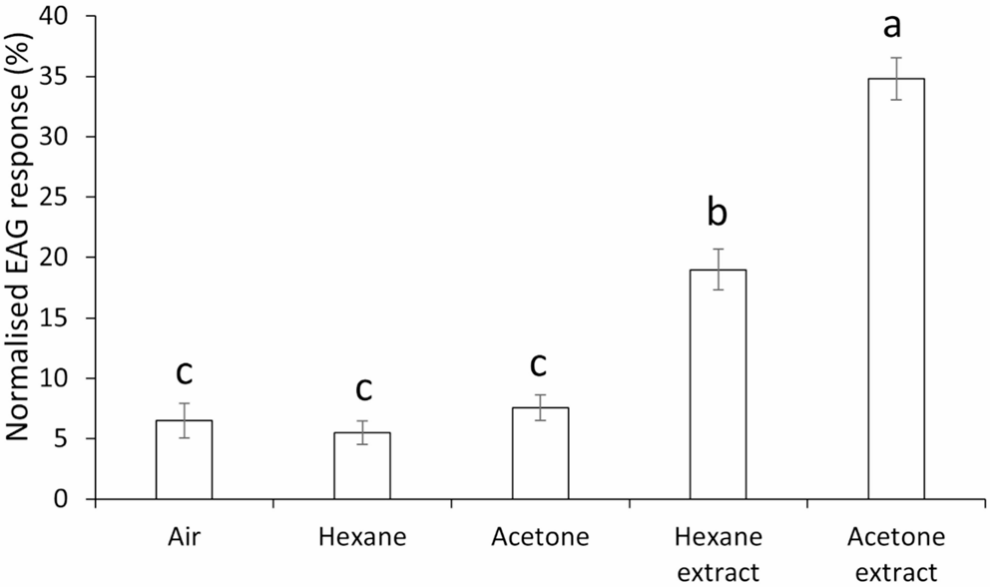
Electroantennogram (EAG) responses of *Trissolcus basalis* to air, hexane, acetone, and *Nezara viridula* egg extracts made with hexane or acetone. EAG responses were normalised against responses to a standard stimulus, (E)-2-decenal. Letters above the bars indicate significant differences by ANOVA followed by Tukey’s HSD test (mean ± SE, *p* = 0.05)

The six electroantennogram recordings, each with a separate antennal preparation, revealed *T. basalis* had significantly higher responses to the acetone extract (*F* = 202.42, df = 4, *P* < 0.001) and hexane extract than corresponding solvent controls, in which the acetone extract exhibited significantly higher EAG responses than hexane extract (Fig. 3). Relative to responses to the standard stimulus, mean normalised responses of *T. basalis* to *N. viridula* extract were 34.8% ± 1.7 (mean ± SE, *n* = 18) for the 96-hour acetone extract and 19.0% ± 1.7 (mean ± SE, *n* = 18) for the 24-hour hexane extract. Air (6.5% ± 1.4, mean ± SE, *n* = 6), hexane solvent (5.5% ± 1.0, mean ± SE, *n* = 18), and acetone solvent (7.6% ± 1.1, mean ± SE, *n* = 18) control treatments elicited comparatively lower responses (Fig. 4).

## Discussion

The combination of arrestment bioassays, competition experiments, and electrophysiology provides much more context for helping to understand the host-specificity of these two parasitoids than any one method alone. We have demonstrated that high parasitism efficiencies (> 90%) reported from oviposition experiments do not necessarily translate into high motivation to search for the host in arrestment experiments. We showed that *T. basalis and T. oenone* displayed very different levels of motivation to search for each host, and that they have very different abilities to compete in both extrinsic and intrinsic contests. We also demonstrated how important the choice of solvent is for preparing egg extracts, and we report a list of potential host acceptance kairomones found on the eggs of *N. viridula*.

*T. basalis* spent longer searching for its primary host *N. viridula*, than for *C. simplex*, while the reverse was true for *T. oenone*. On average, the native parasitoid spent even less time searching for the non-host *N. viridula* than *T. basalis* spent searching for the less preferred host *C. simplex*. These results have two important implications. First, they clearly show that both parasitoids are capable of discriminating between adult hosts based solely on footprint compounds, a fact already known for *T. basalis* and other *Trissolcus* species (Colazza et al. 1999; Conti et al. 2004; Salerno et al. 2006) but as yet undocumented in *T. oenone*. Second, they suggest that *T. oenone* may be able to differentiate between physiological hosts and non-hosts based solely on footprint compound profiles. Peri et al. (2021) showed that naive female *T. basalis* displayed similar arrestment behaviour in arenas contaminated by the suboptimal host *H. halys*, but that parasitoids spent about a third longer in arenas contaminated by their preferred host, *N. viridula*. Parasitoid surveys conducted in Auckland between December 2020 and March 2021 yielded only *T. oenone* specimens in sentinel eggs of *C. simplex* (Pers. Comm., Karina Santos, Plant & Food Research), which may reflect the lack of motivation by *T. basalis* to search for this host as reported here.

We reported the proportion of replicates in which arrestment occurred, as we felt this information was important for assessing the method used to obtain results. While the native parasitoid was arrested in over 70% of replicates for each pentatomid, *T. basalis* was arrested in half of the *C. simplex* replicates, and in just a third of *N. viridula* replicates. The *T. oenone* colony had only been reared through approximately a quarter of the number of generations the *T. basalis* colony had been reared through, so it’s possible the older colony was experiencing decline.

Experiments with other scelionid egg parasitoids have revealed similar discrepancies between no-choice oviposition results and motivation to search in arrestment studies, although they have not been contextualised in terms of their value to pre-release risk assessments. *Telenomus podisi* Ashmead and *Trissolcus urichi* (Crawford) expressed similarly high parasitism rates on *Piezodorus guildinii* (Westood) in no-choice oviposition tests (Cingolani et al. 2014). However, arrestment results showed that, on average, *Tr. urichi* spent about 35% longer searching in arenas contaminated by the host than *Te. podisi* (Cingolani et al. 2019). Another example is provided by Peri et al. (2011), who compared the generalist egg parasitoid *Ooencyrtus telenomicida* (Vassiliev) (Hymenoptera: Encyrtidae) and *T. basalis*, in relation to *N. viridula*. They found that *O. telenomicida* was unable to detect or exploit footprint kairomones associated with *N. viridula*. A subsequent two year field and semi-field experiment in Western Sicily showed *T. basalis* was the superior parasitoid against *N. viridula*, and this is likely to be because *T. basalis* can discriminate between adult *N. viridula* based on their sex and reproductive status (Peri et al. 2006). Arrestment results paired with oviposition data are valuable in the context of pre-release risk assessments, as they can be used to predict whether or not a candidate agent is likely to detect and search for hosts in the field, and the relative amount of search effort expended by the parasitoid for different non-target species.

*Trissolcus oenone* successfully parasitized 21% more host eggs than *T. basalis* during competition assays. This was despite *T. basalis* escalating fights more often, and *T. oenone* having to back down from agonistic interactions more frequently than *T. basalis*. In addition, out of the eggs parasitized by both species, *T. oenone* developed in over 90%. Female *T. basalis* marked a higher proportion of probed eggs, suggesting they may have been more strategic in their oviposition attempts, or interrupted less frequently by *T. oenone*. Overall, *T. basalis* was more aggressive during extrinsic contests on the egg mass, but *T. oenone* achieved more parasitism and clearly dominated the intrinsic contest between parasitoids developing inside the same host egg. Female *T. basalis* foraging on the same egg mass initiated their first aggressive encounters in response to a trade-off between the value of defending unparasitised hosts, and the value of offspring previously deposited into the patch (Field and Calbert 1998). Aggression is typically initiated earlier when host patches are smaller, and when encounter rates and the number of offspring invested are higher. We didn’t measure encounter rate and found no relationship between the onset of aggression and any variables we measured.

We expected the introduced biological control agent *T. basalis* to outperform the native parasitoid in both extrinsic and intrinsic contests, but our results show the native parasitoid is the superior competitor. Cumber (1964) conducted a limited series of competition experiments on several species of pentatomid eggs with *T. oenone* and *T. basalis* (which he referred to as ‘Species N’ and *Asolcus basalis*, respectively). He observed a similar pattern in his experiments where usually several eggs were parasitised before the first aggressive encounter between parasitoids. He reported *T. oenone* to be dominant in both extrinsic and intrinsic contests based on behavioural observations and proportions of emerging parasitoids, but his methods were not defined in detail, lacked sufficient replication, and results were not reported quantitatively. Cumber (1964) observed a relatively common pattern whereby the dominant individual would chase the other away from the egg mass, and then complete oviposition in time to interrupt the renewed intrusion attempt made by the submissive individual. We saw this pattern while observing the two parasitoids on the egg masses and speculate it may offer an explanation as to how *T. oenone* was able to lay eggs in a higher proportion of the host patch than *T. basalis*.

Even when a parasitoid survives the intrinsic contest inside a multiparasitised host egg, it may endure subsequent fitness costs (Cusumano et al. 2016). For example, when *T. basalis* and *O. telenomicida* multiparasitised *N. viridula* eggs, surviving *T. basalis* offspring were smaller, took longer to develop to maturity, and females produced fewer oocytes (Cusumano et al. 2015). Interestingly, *T. basalis* did not experience the same detrimental outcomes after surviving intraspecific competition, whereas the reverse was true for *O. telenomicida*: its offspring suffered similar fitness costs but only as a result of intraspecific and not interspecific competition. This is likely because *O. telenomicida* injects substances which directly alter the nutritional profile of host eggs, and indirectly mediate interspecific competition with other egg parasitoids that multiparasitised eggs it has previously attacked (Cusumano et al. 2012). It is possible that venoms or accessory gland products are injected into host eggs by *T. oenone*, or both parasitoids, and these products may provide *T. oenone* with a developmental advantage over *T. basalis*. Competition experiments are useful for revealing the complex interactions between different parasitoids on the same host. If a candidate agent is less dominant on the egg mass, and especially if it consistently loses the intrinsic contest between larvae, then it is unlikely to displace the native parasitoid.

The significant EAG responses of *T. basalis* to acetone and hexane extracts indicate that the stinkbug eggs produce olfactory-active compounds detected by *T. basalis*, with some polar (extracted in acetone) and non-polar (extracted in hexane) compounds. *Trissolcus basalis* antennal responses were 55% higher to acetone extracts than hexane extracts. Differing immersion times represent a limitation in our work, as we were unable to determine if the stronger responses observed here were due to the solvent or immersion time. However, previous work has shown that acetone extracts of *N. viridula* eggs elicit probing behaviour in *T. basalis* when applied to glass beads, whereas hexane extracts do not (Bin et al. 1993). This suggests acetone effectively removes contact kairomones present in the adhesive used by female stink bugs to glue eggs to each other and onto a substrate. Chemical analysis showed the acetone extract in our study contained far more compounds than the hexane extract, and there was little overlap in compounds between the two extracts.

Michereff et al. (2016) identified a similar blend of compounds in acetone extracts made with *Euschistus heros* (F.), and showed that *Telenomus podisi* Ashmead were attracted to eggs and egg extracts in Y-tube olfactometers. The major compounds identified in *E. heros* extracts were hexadecanoic acid, linoleic acid, octadecenoic acid, octadecanoic acid, and ethyl stearate. This suggests linoleic acid, hexadecanoic acid, and octadecanoic acid make good candidates for contact kairomones as they appear to be common to those found to be attractive to *Te. podisi* (Michereff et al. 2016). However, Tognon et al. (2020) showed *Te. podisi* was attracted to a blend of compounds identified from *E. heros* egg extracts made with a five-minute hexane immersion. Hexane extracts contained none of the compounds we or Michereff et al. (2016) identified as dominant compounds in acetone extracts, but instead provoked behavioural responses with a synthetic blend of camphene, β-pinene, limonene and benzaldehyde. It is possible that pentatomid eggs contain several kairomonal compounds with differing polarities, so that both hexane and acetone each remove some and not others. The extraction and identification of kairomonal compounds is an important step in understanding how chemical ecology influences the host-preferences and host ranges of classical biological control agents, but to date, has not typically been included in pre-release risk assessments.

## Conclusions

Egg parasitoids face the prospect of having to locate and attack a life stage of host which is often cryptic, and whose quality diminishes over a relatively short period of time (Vinson 1998). As a result, these parasitoids have evolved adaptations to perceive and exploit chemical cues which exist on a reliability-detectability spectrum (Turlings et al. 1990; Vet and Dicke 1992; Bin et al. 1993; Colazza et al. 1999): on the one hand, plant volatiles are abundant and easily detectable, but unreliable for conveying information about the presence of hosts; while on the other, host-derived kairomones offer a far more reliable cue to indicate the presence and even the reproductive status of potential hosts, but they are sparser and more difficult to detect.

Regulators often have to make decisions about whether or not to approve the release of a classical agent based solely on laboratory oviposition data (Bigler et al. 2006; van Lenteren et al. 2006; Barratt 2011). A risk assessment for *T. basalis* and *T. oenone* based solely on oviposition data would conclude that both pose the same level of risk to a species such as *C. simplex*, as both parasitoids attacked and emerged in similar numbers from this host (> 90%). However, our arrestment results showed that *T. basalis* is far less motivated to search for *C. simplex* when exposed to substrate-borne footprint compounds in open arenas. We believe arrestment studies have major contributions to make to the study of non-target risks associated with classical biological control agents, as they provide a relatively simple and inexpensive method to provide much-needed behavioural context to complement the results of oviposition tests (Conti et al. 2004). This is particularly useful when oviposition results are similar between different parasitoids, or between different non-target hosts for the same parasitoid. Our competition results showed that a native parasitoid outcompeted an introduced biological control agent in both extrinsic and intrinsic contests. Competition assays provide useful information for understanding how intraguild competition may interfere with the efficacy of a biological control agent on a target pest, or for how adding a new parasitoid to a foodweb may affect non-target species. Finally, electrophysiological experiments are useful for identifying kairomonal compounds responsible for the host preferences expressed by an agent, and we provided a list of candidate compounds which may have wider applicability as kairomones within scelionid-pentatomid systems. Electrophysiology studies are also useful for determining which compounds associated with a host deserve more attention in behavioural experiments, and we think their wider application to pre-release risk assessments would help to improve the way classical biological control agents are screened for potential non-target risks. Oviposition tests, arrestment bioassays, competition experiments, and electrophysiological experiments all provide complementary information on the host-specificity of a candidate agent, and they each provide useful context for interpreting each other.

A combination of chemical and behavioural ecological approaches to host-specificity testing will help to better characterise non-target risks associated with classical biological control agents, as well as to reduce uncertainties that remain after traditional oviposition testing during pre-release risk assessments.

## Data Availability

All data generated during this study is available on Zenodo (https://zenodo.org/doi/10.5281/zenodo.10114949).
